# The Association between the Types of Exercise Motivation and Social Presence about Gerontechnology

**DOI:** 10.1093/geroni/igab046.3349

**Published:** 2021-12-17

**Authors:** Si Young Song, Inhye Jung, Miseon Kang, Kwang Joon Kim, DaeEun Kim, Hey Jung Jun

**Affiliations:** 1 Yonsei University, Seoul, Seoul-t'ukpyolsi, Republic of Korea; 2 Severance Hospital, Yonsei University College of Medicine, Seoul, Seoul-t'ukpyolsi, Republic of Korea

## Abstract

The purpose of this study was to identify the types of exercise motivation and examine the association between the types of exercise motivation and social presence about exercise-related gerontechnology among Korean young-olds. In this study, social presence about gerontechnology implies the degree of perception of a robot that helps exercise functions as human-like socially interacting entities (Heerink, 2010). Online survey data collected from the Korean older adults over the age of 65 in February 2021 was used, and the subjects of this study were 154 young-olds aged 65 to 74 who exercise regularly. Latent class analysis (LCA) was conducted to classify the types of exercise motivation, followed by multiple regression analysis. As a result of LCA, the types of exercise motivation was classified with two groups. These groups were named ‘for pleasure and leisure (PL, 77.2%)’ and ‘for maintenance of health (MH, 22.8%)’, respectively. The result of multiple regression showed that compared to the second group (MH), the social presence about gerontechnology was high for the first group (PL) after controlling age, gender, education level, marital status, household income and chronic disease. These results indicate the Korean young-olds’ exercise motivation may vary and expectations for social presence toward exercise-related gerontechnology differ depending on the exercise motivation. To date, the importance of social presence in gerontechnology has tended to be emphasized mainly in the care field. This study suggests that exercise-related gerontechnology devices also need to consider the aspect of social presence especially for young-olds who exercise for pleasure and leisure.

